# DNA methylation signatures of breast cancer in peripheral T-cells

**DOI:** 10.1186/s12885-018-4482-7

**Published:** 2018-05-18

**Authors:** Surabhi Parashar, David Cheishvili, Niaz Mahmood, Ani Arakelian, Imrana Tanvir, Haseeb Ahmed Khan, Richard Kremer, Catalin Mihalcioiu, Moshe Szyf, Shafaat A. Rabbani

**Affiliations:** 1 Department of Medicine, McGill University Health Center, 1001 Décarie Blvd., Room EM1.3232, Montréal, QC H4A3J1 Canada; 2 Department of Pharmacology and Therapeutics, McGill University Health Center, Montreal, QC Canada; 3 Fatima Memorial Hospital, Lahore, Pakistan; 4 Present address: Montreal EpiTerapia Inc., Montreal, QC Canada

**Keywords:** Breast cancer, Biomarkers, Immune system, DNA methylation, Blood DNA, Epigenetic signature

## Abstract

**Background:**

Immune surveillance acts as a defense mechanism in cancer, and its disruption is involved in cancer progression. DNA methylation reflects the phenotypic identity of cells and recent data suggested that DNA methylation profiles of T cells and peripheral blood mononuclear cells (PBMC) are altered in cancer progression.

**Methods:**

We enrolled 19 females with stage 1 and 2, nine with stage 3 and 4 and 9 age matched healthy women. T cells were isolated from peripheral blood and extracted DNA was subjected to Illumina 450 K DNA methylation array analysis. Raw data was analyzed by BMIQ, ChAMP and ComBat followed by validation of identified genes by pyrosequencing.

**Results:**

Analysis of data revealed ~ 10,000 sites that correlated with breast cancer progression and established a list of 89 CG sites that were highly correlated (*p* < 0.01, *r* > 0.7, *r* < − 0.7) with breast cancer progression. The vast majority of these sites were hypomethylated and enriched in genes with functions in the immune system.

**Conclusions:**

The study points to the possibility of using DNA methylation signatures as a noninvasive method for early detection of breast cancer and its progression which need to be tested in clinical studies.

**Electronic supplementary material:**

The online version of this article (10.1186/s12885-018-4482-7) contains supplementary material, which is available to authorized users.

## Background

Breast cancer is one of the most prevalent malignancies in women affecting as many as one in nine women resulting in a high incidence of morbidity and mortality [1]. An important challenge for effective treatment remains the lack of non-invasive prognostic biomarkers for detection of early-stage breast cancer. Breast cancer is classified based on tumor cells invasive capacity into stages I–IV or according to tumor size (T), lymph node involvement (N) or if it has metastasized (M) to collectively referred to as the TNM staging system of American Cancer Society (https://www.cancer.org). A large body of research spanning almost two centuries has focused on the discovery of sensitive and specific cancer biomarkers [2]. Paul Ehrlich conceived the idea that host immune system can recognize and eliminate tumor cells [2] that was further supported by evidence, demonstrating the ability of the immune system to monitor and eliminate any non-self-antigens or pathogens (reviewed in [3]. According to the immune surveillance theory, that was formulated by Burnet and Thomas [4], immune cells regularly monitor and eliminate arising, nascent tumor cells. T-cells are the most prominent members of the host-immuno-surveillance system, which controls tumor growth [5] and therefore represent an attractive source of cancer biomarkers [6, 7].

The cellular infrastructure of the human body including peripheral immune cells is governed by epigenetic mechanisms which regulate transcriptional machinery [8]. The key role of these epigenetic changes in the detection and monitoring of cancer has been demonstrated in recent years [9, 10]. DNA methylation is one of the most important epigenetic alteration accompanying tumorigenesis [11]. Specific DNA methylation changes in cancer patients white blood cells were demonstrated in head and neck squamous cell carcinoma (HNSCC), in ovarian [12, 13], in colorectal [14], and in hepatocellular carcinoma (HCC) [15]. We have recently demonstrated that the host immune system in HCC has a distinct DNA methylation signature that correlates with HCC progression [16].

In the current study, we tested whether this progressive change in DNA methylation is unique to HCC as previously seen by us which originates from an underlying inflammatory viral disease or it is common to other cancers including breast cancer as well. We used Illumina 450 K arrays to determine the state of methylation of around 450,000 CG sites in the genome of T cells isolated from a cohort of 19 females with early-stage (1 and 2), and nine females of late-stage (3 and 4) breast cancer as well as nine healthy age-matched control healthy females. Hormonal status was not significantly different among patients with different stages of breast cancer and is unlikely to affect the state of methylation among these groups (Additional file 1: Table S1). Our results suggest a large number of CGs that significantly correlate with breast cancer progression supporting the hypothesis of a broad rearrangement of T cell methylome during the progression of breast cancer. The vast majority of these changes involve progressive loss of DNA methylation similar to what was observed in HCC. Importantly, the changes in DNA methylation that correlate with cancer progression are enriched in genes that are involved in immune functions.

## Methods

### Study populations

The study design was approved by the ethics committee of McGill University Health Center (MUHC). Peripheral blood samples from healthy controls and breast cancer patients were obtained from the oncology clinic of MUHC following the approval by the institutional review board (IRB) and written consent was obtained from all control and breast cancer patients. All patients were enrolled at the time of diagnosis before initiation of any treatment including chemotherapy or radiotherapy. Detailed information about the breast cancer cases and controls is shown in Additional file 1: Table S1.

### T-cell isolation

CD3+ T cells were isolated from 8 ml blood drawn from age matched control women and women at different stages of breast cancer using CD3 dynabeads (Life Technologies, Toronto, Ontario, Canada). Following the extraction of T cells, DNA was extracted using AllPrep DNA/RNA mini kit (Qiagen, Canada) and whole genome DNA methylation profiles were generated using Illumina 450 K bead arrays.

All the peripheral whole blood samples were stored in EDTA tubes at 4 °C until Leukocyte isolation. Leukocytes were freshly isolated from whole blood by using ficoll gradient separation. The leukocyte cell pellets were immediately frozen at − 80 °C until further use. First, B cells were positively isolated using a Dynabeads CD19 positive isolation kit (Invitrogen). Subsequently, these B cell-depleted leukocytes were used for T-cell purification with a Dynabeads CD3 positive isolation kit (Invitrogen). The T-cell pellets were immediately frozen at − 80 °C for further DNA isolation. DNA was isolated from different blood cell types using AllPrep DNA/RNA Mini Kit from Qiagen.

### Illumina 450 K methylation analysis

Genomic DNA from all the breast cancer cases and controls was quantified using Picogreen protocol (Quant-iTTM PicoGreen_ dsDNA Products, Invitrogen, P-7589) and read on a Spectra-MAX GeminiXS Spectrophotometer. Bisulfite conversion of 500 ng of genomic DNA was performed using the EZ-96 DNA Methylation-GOLD Kit (Zymo Research, Irvine, CA, USA). The Illumina Methylation 450 K kit (San Diego, California, USA) was used for the microarray experiment as described by the manufacturer’s protocol, except that 8 uL of bisulfite converted template was utilized to initiate the amplification step. The Illumina hybridization oven was used for incubating amplified DNA (37 °C) and for BeadChips hybridization (48 °C).

A Hybex incubator was used for fragmentation (37 °C) and denaturation (95 °C) steps. The X-stain step was carried out in a Tecan Freedom evo robot with a Te-Flow module. Arrays were scanned in Illumina iScan Reader.

### Statistical analysis

The raw data obtained from the Illumina 450 K arrays were processed from the IDAT files through to normalization with BMIQ [17] using the ChAMP [18] pipeline, batch correction for technical replication dataset using ComBat [19] and all subsequent analyses were performed with the R statistical software v3.2.1.

Quality control of the array data included removal of 2394 probes for which any sample did not pass a 0.01 detection *P*-value threshold, filtering probes with a bead count less than 3 has removed 267 probes from the analysis. Filtering probes with single nucleotide polymorphism (SNPs) as identified in Nordlund et al. [20], removed 28,391 probes from the analysis. We corrected for multiple testing using Benjamini-Hochberg False Discovery Rate (FDR) correction, our significance threshold was set at adjusted *p* value *q* < 0.05. Filtering probes that align to multiple locations as identified in Nordlund et al., has removed 8482 probes from the analysis. The Kaplan-Meier relapse-free survival plots were generated by KM-Plotter [21].

### Pyrosequencing analysis

Genomic DNA (200–500 ng) was used for bisulfite conversion using the EZ-DNA methylation Gold Kit (Zymo Research, Irvine, CA, USA). Pyrosequencing validation of selected genes was performed (See Additional file 1: Table S2 for list of primers used). The number of genes subjected to pyrosequencing was limited by the amount of DNA obtained from these clinical samples. Samples were prepared by performing PCR amplification of selected CGs. PCR reactions were conducted using Hot star enzyme in Biometra T Gradient and T3 thermocyclers. Pyrosequencing was performed using standard methods; briefly, biotinylated PCR products were incubated with streptavidin sepharose beads (GE Healthcare, Canada), followed by denaturation. Beads containing the biotinylated strand were released into 25 μl annealing solution and 0.3 mM sequencing primer per well. Pyrosequencing was performed using PyroMark Q24 and results were analyzed with PyroMark® Q24 Software (Qiagen, Toronto, Ontario, Canada). Collected data was expressed as mean ± standard error of the mean (SEM) and using Student’s t-test, *p*-value< 0.05. The statistical analysis was performed using Prism (GraphPad Software Inc., San Diego, California).

## Results

DNA was isolated from breast cancer patients (19 females of breast cancer stages 1 and 2, five with stage 3 and four with stage 4) compared to 9 age-matched healthy females (*p* = 0.5, t-test) (Additional file 1: Table S1) according to the staging criteria of American Cancer Society (https://www.cancer.org). Genomic DNA from T cells was analyzed using Illumina Infinium HumanMethylation450 BeadChip arrays [22]. Raw data from all samples was analyzed using the ChAMP analysis pipeline [18] that included BMIQ normalization [17] and ComBat function which corrects batch effects related to BeadChip. Differentially methylated CGs were called using Bioconductor package Limma [23] as implemented in ChAMP using FDR for multiple testing correction (adjusted *P* value (Q) of < 0.05). After filtering probes that did not pass primary quality control SNPs and repetitive probes (see Methods), the analysis proceeded with 445,978 CpG probes. Almost all breast cancer patients were estrogen and progesterone receptor positive and HER2 receptor negative (for clinical characteristics see Additional file 1: Table S1). To exclude confounding clinical factors involvement in DNA methylation we performed linear regression analysis for age or hormonal status (ER, PR, and HER2). These confounding factors (except one CG site that was correlated with progesterone) showed no correlation with average methylation values across the group.

### Site-specific DNA methylation levels correlates with progression of breast cancer

**Fig. 1:**
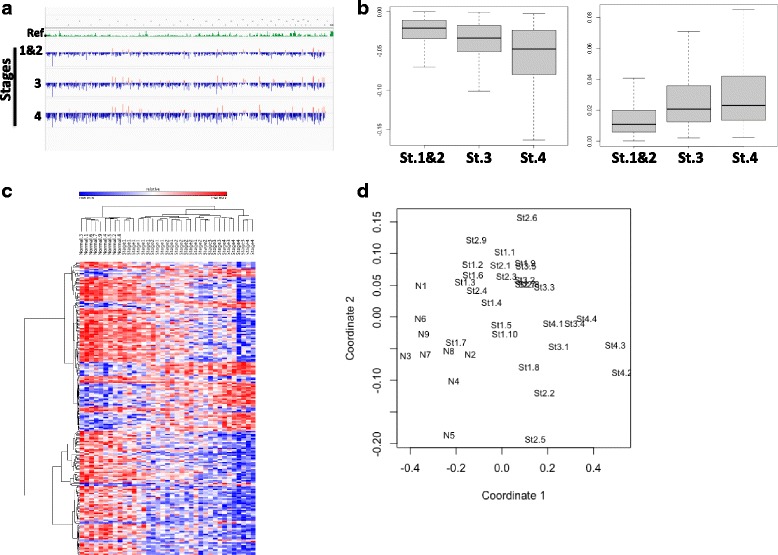
**a** Genome wide distribution of 10,772 CpGs whose DNA methylation progressively changes with breast cancer progression from stages 1 to 4. **b** Boxplot of 8238 significantly (*p* value< 0.05) demethylated (left panel) and 2408 hypermethylated (right panel) CpGs associated with breast cancer progression (delta value between breast cancer stages and average beta values of all normal individuals) in stages 1, 2, 3 and 4 in breast cancer. **c** Heatmap and hierarchical clustering of top 89 CpG whose quantitative level of DNA methylation correlate with progression (*p* < 0.01, *r* > 0.7, *r* < − 0.7). **d** Principal component analysis of unaffected females (N1-N9), females with breast cancer stage 1(St1.1-St1.10), stage 2 (St2.1-St2-9), stage 3 (St3.1-St3.5) and stage 4 (St4.1-St4.4) methylation profiles, plot show principal component 1 (coordinate 1) and principal component 2 (coordinate 2) for each sample. Close to each other samples are similar in their methylation profile

We performed Pearson correlation analysis (Hmisc R) to determine whether DNA methylation changes in T cells correlate with breast cancer progression [23, 24]. This analysis revealed statistically significant (*p* < 0.05) 10772 CpG sites whose DNA methylation level correlate with breast cancer progression; 8283 of them were hypomethylated and 2489 hypermethylated (Additional file 1: Table S3). A genome-wide view of 10,772 CpG sites that demonstrate progressive methylation changes associated with breast cancer stage is represented in Fig. 1a. A boxplot of DNA methylation mean delta values (difference between average DNA methylation of cancer patients and healthy females) demonstrate intensifying (Fig. 1b, hypomethylation-left panel, and hypermethylation right panel) hypomethylation of DNA with breast cancer progression. One-Way ANOVA test revealed significant differences in the overall average methylation of these 10,772 CpGs between different breast cancer stages and normal individuals (between normal and stages 1 and 2, *p* < 0.01, between normal and stage 3, *P* < 0.00001 and between normal and stage 4, *P* < 0.00001). These data support the hypothesis of a broad change in DNA methylation of T cells as cancer progresses which is similar to what was recently observed in HCC [16].

Heat map and hierarchical clustering analysis (Pearson minus one correlation) of the most significant 89 CpG sites (*p* < 0.01, *r* > 0.7, *r* < − 0.7) (Additional file 1: Table S4), whose DNA methylation levels correlate with breast cancer progression, grouped the early 1 and 2 stages and late (3 and 4) separately from each other, suggesting that the combination of these sites predicts breast cancer stages in T cells accurately across individuals (Fig. 1c, d). Multivariate linear regression showed that these CG sites remained significant even when age and hormonal status were included in the model.

**Fig. 2:**
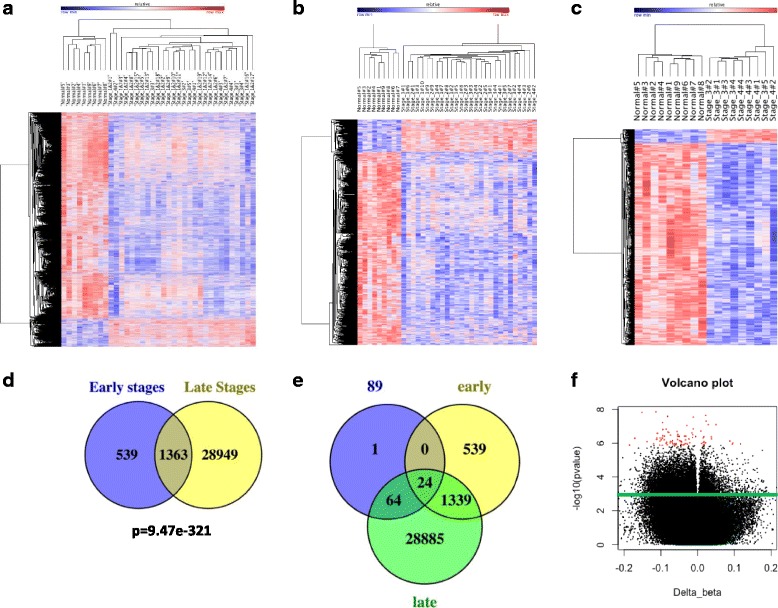
Differentially Methylated CG Sites at different stages of breast cancer patients. **a** Heat map of hierarchical clustering of nine healthy individuals and 28 breast cancer patients by beta values of 10,859 differentially methylated CGs (*p* < 0.05). **b** Heat map of hierarchical clustering of 1902 differentially methylated CGs (*p* < 0.05) in early stages of breast cancer, in healthy individuals and early and late stages of breast cancer patients. **c** Heat map of hierarchical clustering of nine healthy individuals and 3 and 4 breast cancer stages 9 patients by beta values of top 2239 differentially methylated CGs (*p* < 0.01). **d** Venn diagram showing significant overlap (*p* = 9.47e-321, hypergeometric) of methylation changes between early (1 and 2) and late (3 and 4) stages. **e** Venn diagram showing overlap of top 89 CpG whose quantitative level of DNA methylation correlate with progression with differentially methylated CpGs in early and late stages of breast cancer. **f** Red dots indicate 89 the most significant CpG sites (adjusted *P* value< 0.05, and *R* > 0.7 or *R* < 0.7), whose DNA methylation level correlate with breast cancer progression. Delta beta indicates the differences of DNA methylation between average of stage 4 and normal individuals. Green line separate between 10,772 CpG sites (top) whose DNA methylation level correlate with breast cancer progression and not significantly changed CpGs DNA methylation (bottom)

We also used a case control design and a mixed linear regression LIMMA [23] to determine association between methylation state and presence of breast cancer. First, we found 10,859 FDR adjusted significantly differentially methylated CGs (9564 hypomethylated and 1295 hypermethylated) (Fig. 2a) between healthy females and all breast cancer patients (*p* < 0.05) (Additional file 1: Table S5). Heatmap and hierarchical clustering analysis of these sites accurately grouped all cancer patients away from normal, suggesting that the DNA methylation profile of T cells associates with breast cancer (Fig. 2a).

We next compared separately the differences in DNA methylation between early stages (*n* = 19) and healthy controls (*n* = 9) (Fig. 2b, Additional file 1: Table S6) and between late stages (*n* = 9) and healthy controls (*n* = 9) (Fig. 2c, Additional file 1: Table S7). Similar to the results of the Pearson correlation analysis across all individuals (Fig. 1a, b, c), the case control analyses revealed progressive loss of DNA methylation with advanced breast cancer stage. Specifically, early breast cancer stages were associated with 1902 differentially methylated probes (1629 hypomethylated and 273 hypermethylated probes) spanning 1590 genes (Fig. 2b, Additional file 1: Table S6), while the late breast cancer stages were associated with 30,312 differentially methylated probes (27,049 hypomethylated and 3263 hypermethylated) spanning 12,705 genes (Fig. 2c, Additional file 1: Table S7). These results are particularly interesting, considering the fact that the sample size, that has direct effect on statistical power, was much larger when we compared early stages (*n* = 19) with controls, than we compared late stages (*n* = 9) to controls. The fact that both analyses reveal the same progressive broad change in methylation as breast cancer advances further support the idea of a distinct DNA methylation profile in T cells in breast cancer.

Interestingly, although analyzed separately there was a very significant overlap of 1363 probes (Fig. 2c) (Additional file 1: Table S8) (*P* = 9.47e-321, hypergeometric) between CG sites that were differentially methylated from healthy controls at early stages and late stages as can be seen in heatmap presented in Fig. 2b further validating the significance of these sites.

Importantly, the top 89 CpG sites (Fig. 1c) (*p* < 0.01, *r* > 0.7, *r* < − 0.7), whose DNA methylation level correlates with breast cancer progression and differentially methylated sites in both early and late breast cancer stages completely overlapped (except one CG site) (Fig. 2e). The most significant 89 CpGs which correlate with breast cancer progression (Additional file 1: Table S4) are also shown as a volcano graph (Fig. 2f).

### Differentially methylated genes are enriched with immune functions

To assess which gene networks, functional categories, and canonical pathways undergo DNA methylation alterations in T cells in breast cancer patients we used the Ingenuity Pathway Analysis (IPA) tool. Table 1 and Additional file 1: Table S9 show the detailed list of canonical pathways of differentially methylated genes in T cells of breast cancer. Remarkably, the top significant canonical pathways include, T helper cell differentiation (*p* = 2.45E-05) and Altered T cell and B cell signaling in Rheumatoid Arthritis (*P* = 4.67E-05). Top upstream regulators include lipopolysaccharide (*p* = 6.6e-13), TNF (*p* = 7.8e-13), TGFB1 (6.2e-11) and immunoglobulin (1.73e-10) major regulator of immune cells function (Additional file 1: Table S10). These data support the hypothesis that the changes in DNA methylation in T cells are associated with the immune system of the host organism and not with DNA methylation occurring in the cancer cells.

**Table 1:** Ingenuity canonical pathways analysis

Ingenuity canonical pathways	*p*-value
Type I Diabetes Mellitus Signaling	5.8884E-06
T Helper Cell Differentiation	2.4547E-05
CDP-diacylglycerol Biosynthesis I	2.6303E-05
Altered T Cell and B Cell Signaling in Rheumatoid Arthritis	4.6774E-05
Phosphatidylglycerol Biosynthesis II (Non-plastidic)	4.6774E-05
Hematopoiesis from Pluripotent Stem Cells	7.4131E-05
Hepatic Cholestasis	1.7378E-04
Dendritic Cell Maturation	1.7783E-04

### Validation of DNA methylation obtained from Illumina 450 K by pyrosequencing

**Fig. 3:**
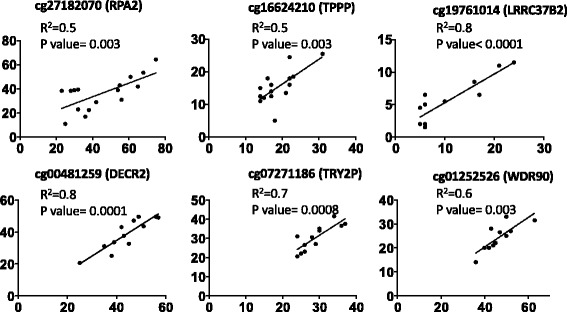
Validation of Illumina 450 K DNA methylation bead array by Pyrosequencing. Correlations between Illumina 450 K array data and pyrosequence analysis. Representative data for CpG sites in cg27182070 (*RPA2*), cg16624210 (*TPPP*), cg19761014 (*LRRC37B2*), cg00481259 (*DECR2*), cg07271186 (TRY2P), cg01252526 (*WDR9*), and genes is shown

We determined a correlation between DNA methylation levels obtained from Illumina 450 K and pyrosequencing. Pyrosequencing analysis was limited by the remaining amount of T-cell DNA. A few representative samples were used for validation purposes. Nine normal and five breast cancer T-cell DNA samples were subjected to bisulphite conversion and pyrosequencing analysis. Seven CG probes that varied across the samples were randomly selected. Figure 3 shows the correlation between values obtained by Illumina analysis and pyrosequencing for these CGs. Correlations were significant for all probes with *r* values between 0.5 and 0.8 (Fig. 3).

### Association of identified gene panel with breast cancer relapse free survival

**Fig. 4:**
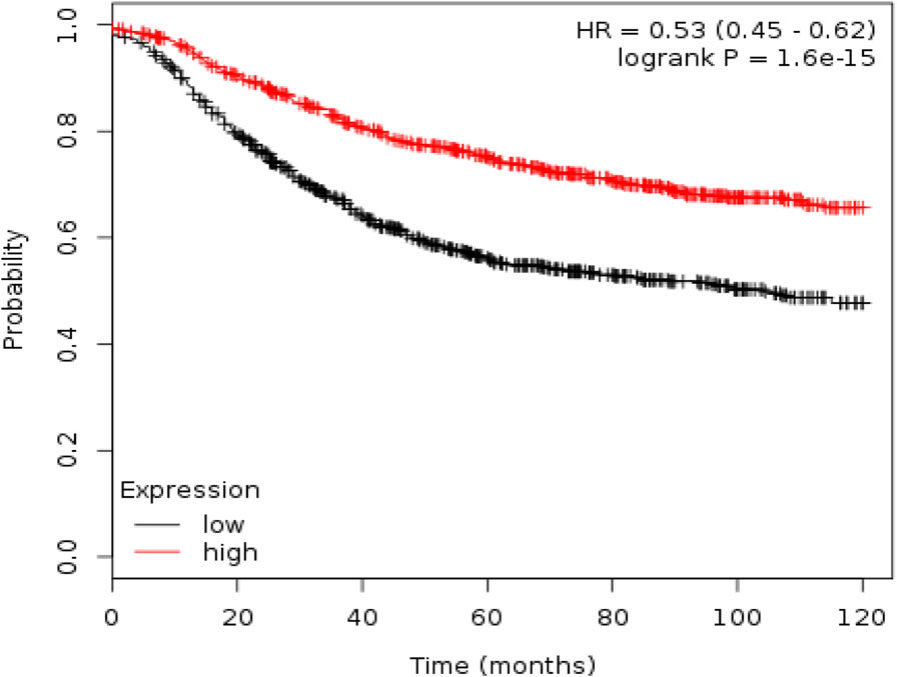
Association of identified gene panel with disease-free survival. Kaplan-Meier survival curve generated from the combined expression of the identified panel of genes shows strong association between the higher expression of these genes with breast cancer patients relapse-free survival

Identified gene panel shown in Fig. 3 was further analyzed using the KM plotter (Kaplan-Meier plotter) database of breast cancer patients in order to investigate the prognostic significance of the genes in breast cancer relapse-free survival. The KM-plotter database was generated by Gyorffy et al. using the NCBI Gene Expression Omnibus (GEO) repository of gene expression and patient survival information [21], and is often used to investigate the clinical significance of particular gene(s) in several common cancers. Using gene expression data from 1764 breast cancer patients, an association between the decreased expression of genes identified and validated in Fig. 3 was observed with a lower incidence of relapse-free or disease-free survival (Fig. 4). The high or low expression groups were classified according to whether the combined expression of the genes was greater than their median expression.

## Discussion

Aberrant DNA methylation is one of the hallmarks of cancer tissue. However, less is known about the alterations occurring in DNA methylation in non-cancer tissues in cancer patients. DNA methylation of peripheral blood cells in cancer might have potential as a diagnostic tool. Our data is consistent with the idea that DNA methylation alterations occur in peripheral T cells that correlate with cancer progression.

We hypothesize, that DNA methylation analysis of T cells can also be applied for detection of early stages of breast cancer. Though, various biomarkers for breast cancer have been proposed, early diagnosis of breast cancer is still a challenge [25, 26]. The current imaging methods are also restricted by the size and volume of growing tumor tissue [27]. Mostly, current methods of breast cancer detection depend on invasive methods like biopsy of tumor tissue [28]. Early detection of breast cancer before the appearance of tumors, could improve breast cancer diagnosis and prognosis.

Cancer cells frequently escape the immune surveillance mechanisms and disseminate to newer sites for metastasis. These metastatic cancer cells are epigenetically programmed to alter the genetic machinery and establish themselves in the favorable environment. The peripheral cells of the immune system constantly patrol the body to protect it from pathogens, exogenous antigens and are able to identify the transformed cells [27]. T-cells are involved in cancer immune surveillance [28, 29]; deregulation of their role is therefore hypothesized to be involved in cancer progression. Since phenotypic alterations are associated with epigenetic changes it is hypothesized that progression of cancer is associated with alteration of DNA methylation in T-cells. In the present study we show extensive alterations in T cells from breast cancer patients that are associated with progression of breast cancer. The genes that are differentially methylated are enriched in immune functions, which is consistent with the hypothesis that these alterations in DNA methylation in T cells are associated with functional changes which might in turn be involved in progression of breast cancer.

A list of 89 CGs clusters all individuals by their cancer stage, pointing to the possibility of using a combination of CG methylation states to detect and stage breast cancer early noninvasively. It is important to note that these CGs detect early stages of breast cancer and no association was found with allergy, immune mediated disorders or inflammation [30, 31]. Genes validated in Fig. 3 are involved in DNA replication and repair, cell cycle, mitosis, oligodendrocyte differentiation, tubulin polymerization, signal transduction, transcription regulation, autophagy, apoptosis and regulation of lipid metabolism, which collectively play an important role in several malignancies including breast cancer. The uniqueness of the identified signatures was further confirmed by the survival-curve generated from their gene expression profile in breast cancer (Fig. 4). Future prospective clinical studies are required to determine whether they can detect breast cancer earlier than currently available imaging methods. In these cases, loss of DNA methylation associated with advanced breast cancer stage may in fact reflect the demethylation of pro-metastatic genes as previously described by us [32–35]. Results from these studies are in line with identified CpG probes which showed a change in DNA methylation and correlation with disease progression in liver cancer patients [16]. We also observed overlap of probes among DCIS, mixed and invasive breast cancer, their association with canonical pathways and upstream regulators of gene expression (Additional file 1: Tables S11–S13).

We next compared data from our study to previously reported epigenome wide association studies (EWAS) which examined the risk of breast cancer development using whole blood [36–41]. In these prospective cohort studies no significant overlap between CpGs differentially methylated in T-cells from our study and 250 differentially methylated CpGs at FDR threshold < 0.05 was found [37]. Interestingly however, the majority of the probes from previously reported study were hypomethylated in breast cancer cases compared with controls that correspond to the results of our study (Fig. 2) [37]. Comparison of differentially methylated probes from our study to the top ranked 2514 CpGs in white blood cells associated with BRCA1 mutation showed significant overlaps between these sites and the sites differentially methylated in the late stages (*P* = 3e-22) and the sites differentially methylated in early stages (*P* = 2.3e-06) in our study [36]. One of the limitations of this pilot study is the small size of the groups. Nevertheless, cross validation comparing the sites that are differentially methylated between stage 1 and 2 and healthy controls and sites that are differentially methylated between late stage and controls shows a significant overlap. This analysis also reveals intensification of the differences in methylation from controls in the late stages similar to the results of the correlation analysis. Since no information regarding the current and past smoking history of our patients was available, smoking was not included as a covariate in the model which is a limitation of our data. A larger follow up study should include smoking data since tobacco smoke is reported to alter the methylation state of tumoral DNA [42]. Using our current cohort, the number of samples did not allow sufficient power of analysis based on TNM staging. We are pleased that with our current subjects we were not only ably to differentiate normal women from breast cancer but also among early and late stages of breast cancer. Our stated objective is to use a larger cohort that will allow us to evaluate differences based on stage, TNM and among various breast cancer subtypes as well.

Our goal remains to carry out follow up studies in a larger cohort of breast cancer patients at different stages with representation of various subtypes using T cells. These studies will lead to the identification of an epigenetic signature in T cells that will be strong, specific and will reflect early changes in immune cells. We anticipate that we will be able to shortlist a small number of DNA methylation sites that could serve as a polygenic DNA methylation marker of breast cancer and breast cancer stage. Such an assay could be easily performed with high throughput multiplexed methylation assay and will be analyzed by a streamlined model for prediction of cancer that is based on the combined weight of the methylation levels of the few sites included in the polygenic marker. We also anticipate the signal to be robust enough to be detected in white blood cell DNA that will be simple to use for large scale screening and monitoring of women at risk, prognosis and designing therapeutic strategies including epi-drugs currently under development.

## Conclusions

Our study provides justification for further exploring the possibility that differential DNA methylation plays a role in T cell function in breast cancer and that it might serve as a biomarker for noninvasive early detection of breast cancer. Correlation of the levels of T cell DNA methylation can be made with TNM staging in breast cancer that can result in the development of a molecular signature of immune staging. Further studies with a larger number of samples are required to address this question.

## Additional file

Additional file 1: **Table S1.** Clinical table of normal individuals and cancer patients. **Table S2.** Primer sequences. **Table S3.** List of CpG probes, whose DNA methylation changes correlate with progression in t-cells of breast cancer patients. **Table S4.** List of top 89 CpG probes (*r* > 0.7, *r* < − 0.7, *p* < 0.01), whose DNA methylation changes correlate with progression in t-cells of breast cancer patients. **Table S5.** Differentially methylated probes in t-cells of breast cancer patients. **Table S6.** Differentially methylated probes in t-cells of breast cancer patients (stages 1 and 2). **Table S7.** Differentially methylated probes in t-cells of breast cancer patients (stages 3 and 4). **Table S8.** List of overlapped differentially methylated probes between stages 1,2 and stages 3 and 4 in t-cells of breast cancer patients. **Table S9.** Ingenuity Canonical Pathways of differentially methylated genes in T cells of breast cancer. **Table S10.** Upstream regulators of differentially methylated genes in T cells of breast cancer. **Table S11.** Overlap CpG probes, whose DNA methylation changes correlate with progression in t-cells of breast cancer patients and differentially methylated probes in DCIS, mixed and invasive breast from dataset GSE60185. **Table S12.** Canonical Pathways of genes whose DNA methylation changes with breat cancer progression in T cells and overlapped with differentially methylated genes in DCIS, mixed and invasive breast cancer. **Table S13.** Upstream regulators of genes whose DNA methylation changes with breat cancer progression in T cells and overlapped with differentially methylated genes in DCIS, mixed and invasive breast cancer. (XLSX 6740 kb)

## Abbreviations

ER: Estrogen receptor
GEO: Gene expression omnibus
HCC: Hepatocellular carcinoma
HER2: Human epidermal growth factor receptor 2
HNSCC: Head and neck squamous cell carcinoma
IRB: Institutional review board
MUHC: McGill University Health Center
PBMC: Peripheral blood mononuclear cells
PR: Progesterone receptor
